# Can Community Health Workers Report Accurately on Births and Deaths? Results of Field Assessments in Ethiopia, Malawi and Mali

**DOI:** 10.1371/journal.pone.0144662

**Published:** 2016-01-05

**Authors:** Romesh Silva, Agbessi Amouzou, Melinda Munos, Andrew Marsh, Elizabeth Hazel, Cesar Victora, Robert Black, Jennifer Bryce, RMM Working Group

**Affiliations:** 1 Institute for International Programs, Johns Hopkins Bloomberg School of Public Health, Baltimore, Maryland, United States of America; 2 Economic and Social Commission for Western Asia, United Nations, Beirut, Lebanon; 3 Division of Data, Research and Policy, UNICEF, New York, New York United States of America; 4 Universidade Federal de Pelotas, Pelotas, Brazil; The George Washington University School of Medicine and Health Sciences, UNITED STATES

## Abstract

**Introduction:**

Most low-income countries lack complete and accurate vital registration systems. As a result, measures of under-five mortality rates rely mostly on household surveys. In collaboration with partners in Ethiopia, Ghana, Malawi, and Mali, we assessed the completeness and accuracy of reporting of births and deaths by community-based health workers, and the accuracy of annualized under-five mortality rate estimates derived from these data. Here we report on results from Ethiopia, Malawi and Mali.

**Method:**

In all three countries, community health workers (CHWs) were trained, equipped and supported to report pregnancies, births and deaths within defined geographic areas over a period of at least fifteen months. In-country institutions collected these data every month. At each study site, we administered a full birth history (FBH) or full pregnancy history (FPH), to women of reproductive age via a census of households in Mali and via household surveys in Ethiopia and Malawi. Using these FBHs/FPHs as a validation data source, we assessed the completeness of the counts of births and deaths and the accuracy of under-five, infant, and neonatal mortality rates from the community-based method against the retrospective FBH/FPH for rolling twelve-month periods. For each method we calculated total cost, average annual cost per 1,000 population, and average cost per vital event reported.

**Results:**

On average, CHWs submitted monthly vital event reports for over 95 percent of catchment areas in Ethiopia and Malawi, and for 100 percent of catchment areas in Mali. The completeness of vital events reporting by CHWs varied: we estimated that 30%-90% of annualized expected births (i.e. the number of births estimated using a FPH) were documented by CHWs and 22%-91% of annualized expected under-five deaths were documented by CHWs. Resulting annualized under-five mortality rates based on the CHW vital events reporting were, on average, under-estimated by 28% in Ethiopia, 32% in Malawi, and 9% in Mali relative to comparable FPHs. Costs per vital event reported ranged from $21 in Malawi to $149 in Mali.

**Discussion:**

Our findings in Mali suggest that CHWs can collect complete and high-quality vital events data useful for monitoring annual changes in under-five mortality rates. Both the supervision of CHWs in Mali and the rigor of the associated field-based data quality checks were of a high standard, and the size of the pilot area in Mali was small (comprising of approximately 53,205 residents in 4,200 households). Hence, there are remaining questions about whether this level of vital events reporting completeness and data quality could be maintained if the approach was implemented at scale. Our experience in Malawi and Ethiopia suggests that, in some settings, establishing and maintaining the completeness and quality of vital events reporting by CHWs over time is challenging. In this sense, our evaluation in Mali falls closer to that of an efficacy study, whereas our evaluations in Ethiopia and Malawi are more akin to an effectiveness study. Our overall findings suggest that no one-size-fits-all approach will be successful in guaranteeing complete and accurate reporting of vital events by CHWs.

## Introduction

Work to develop plans for monitoring public health and the new Sustainable Development Goals is at the top of the international public health agenda [1,2]. As a part of these efforts, there are increasing demands for measurement of short-term changes in mortality among children less than five years of age in low- and middle-income countries. In most of these countries, health information systems are weak, and Ministries of Health and their partners must rely on household surveys as the primary source of data on vital events and rates [3]. These surveys are conducted only every three to five years in most countries, and produce rates of under-five mortality that are three or more years out of date [4, 5].

The routine collection of vital events data is difficult in rural areas of low-income countries. Most births and deaths do not occur in health facilities, and thus reliance on facility-based vital events data often results in systematic under-estimation of under-five mortality rates [6]. The passive nature of traditional vital registration systems in rural settings of low-income countries, whereby individuals must travel long distances to register an event in areas where transportation and communication infrastructure is often rudimentary, has also led to problems in the timing, accuracy and relevance of vital statistics constructed from civil registration systems [7]. Recently, the United Nations Statistics Division (UNSD) and World Health Organization (WHO) reported that in sub-Saharan Africa, only 7% of births and 6% of deaths are registered by official civil registration systems [8]–thus such birth and death registration systems are not presently viable systems for the monitoring of short-term changes in child mortality rates.

The importance of ‘real-time’ mortality measurement to advance public health has a long and rich history, dating back to the times of John Graunt in 16^th^ century London [9]. In recent years, advocates have promoted two principal approaches to improve and scale up civil registration and vital statistics (CRVS) systems in low-income countries: the development of sample registration systems (SRSs); and the scaling-up of health and demographic surveillance systems (HDSSs). Sample registration involves the routine collection of vital events in a geographically representative sample of the national population. SRSs are currently the primary source of vital statistics in India [10] and China [11], and have been introduced in Indonesia [12], Tanzania [13], Zambia, and Vietnam [14]. HDSS sites have remained small, and are not generally designed to improve CRVS at country level. However, Ye (2012) has advocated for the introduction of multiple, strategically-located HDSSs throughout a given country as an interim measure to monitor mortality over time [15]. Mercer et al. (2014) have substantially expanded this idea by proposing small-area estimation and spatio-temporal smoothing approaches that integrate HDSS data with available sample surveys to produce estimates of child mortality over time [16]. Both of these approaches incorporate active, routine collection of data on vital events, rather than relying on passive systems of vital events reporting by the local population. However, a notable shortcoming of these approaches is that both the development of SRSs and the expansion HDSSs require the setting up of new, parallel, data systems that are separate from existing health systems and the formal CRVS.

Recently in a number of low-income countries, including Ethiopia, Malawi, India, and Pakistan, the role of CHWs has been substantially expanded from provision of community awareness and disease prevention to include provision of safe delivery and integrated community case management of preventable diseases with the highest child mortality burdens (such as malaria, pneumonia, malnutrition, and diarrhoea) [17, 18]. Thus CHWs create a bridge between providers of health, social and community services and communities that may have difficulty in accessing these services [19]. Further, in settings such as Malawi, local communities have reported favorable impressions of community-based health workers [20]. Hence, in theory, CHWs are well-placed to proactively track pregnancies as well as document births and childhood deaths, given that a core focus for CHWs in many low-income countries is maternal, newborn and child health. In that vein, this study explores the viability of CHW-based reporting of births and childhood deaths as an interim measure to advance real-time measurement of under-five mortality and scale-up of CRVS in low-income countries.

In this paper, we synthesize findings from a multi-country study that developed, implemented and evaluated community-based methods to measure changes in under-five mortality for recent periods of 12 months or less at small-scale. This study is part of a broader effort to advance the measurement and accountability agenda for maternal and child health by testing and developing new methods for estimating under-five mortality in “real-time”, i.e., for recent periods of 12 months [21]. We studied the completeness and accuracy of vital events reporting by CHWs in Ethiopia, Ghana, Malawi, and Mali. In particular, we assessed the accuracy of annualized child mortality rates derived from vital events reporting by these community-based health workers by comparing them to annualized child mortality rates derived from full pregnancy or full birth history data collected via household surveys or censuses, and assessed the running costs of implementing each method. Reporting from Ghana has been delayed due to concerns about data quality, and will be added to the Collection at a later date if these concerns are able to be addressed.

## Methods

### Ethics Statement

Ethical clearance for the project was obtained in the United States from the Johns Hopkins Bloomberg School of Public Health (JHSPH)'s Institutional Review Board, in Ethiopia from the Oromia Regional Health Bureau, in Mali from the Ethical Review Committee of the University of Bamako, and in Malawi from the National Health Sciences Research Committee. For each household survey, oral informed consent was obtained from each participant. Consent forms were translated into local languages: Amharic in Ethiopia, Chichewa in Malawi, and Bambara and French in Mali. The IRB at JHSPH waived the need for written consent from the study participants given the low literacy of the population under study. Approval letters are available upon request.

### Setting and Selection of ‘Real-time’ Monitoring of Under-five Mortality (RMM) areas

We selected the countries that would be invited to participate in the RMM project in March 2008. These countries included Ethiopia, Malawi, and Mali; further details on the selection process are available elsewhere [21]. Working with the Ministry of Health in each country, we selected partner institutions. Our in country research partners were Miz Hasab Research Center and the Alliance for Better Health Services PLC (ABH) in Ethiopia, the National Statistical Office of Malawi, and CREDOS in Mali.

Ethiopia, Malawi and Mali are low-income countries in sub-Saharan Africa and are amongst the countries with the poorest health indicators in the world. As shown in Table 1, we purposefully chose small areas with relatively high under five mortality rates and high total fertility rates.

**Table 1:** Demographic Characteristics of RMM Study Sites.

Country	RMM Study Site Areas	Estimated Population Size of Districts/	Estimated Population Size of RMM Study Area	Under-five Mortality Rate (U5MR)	Total Fertility Rate (TFR)
**Ethiopia**	Jimma, West Hararghe	4.4 million (2007 census)	509,395	122 (Oromia region, 2005 DHS)	6.2 (Oromia region, 2005 DHS)
**Malawi**	Balaka, Salima	657,075 (2008 census–both districts combined)	203,741	160 (Balaka) 144 (Salima) (district estimates from 2006 MICS)	6.3 (Balaka) 7.1 (Salima) (district estimates from the 2006 MICS)
**Mali**	Barroueli, Niono	568,993 (2009 census)	32,128 (RMM census)	262 (regional estimate from 2006 DHS)	7.1 (regional estimate from 2006 DHS)

### Formative research

We conducted cross-sectional qualitative assessments in each study location prior to launching RMM activities, to understand current practices for recording vital events, actors involved in the recording of vital events, and barriers and local attitudes towards recording vital events data. The methods and results of this research are described in related articles [22, 23, 24].

### Selection of cadre of community-based workers

We used the results of the formative research in each site as a basis for selecting the community health worker cadre responsible for RMM reporting. Table 2 summarizes the characteristics of CHWs across our three study sites. In all three of these settings, vital events reporting was part of the formal job description of the respective CHWs, although they had other health-related responsibilities as well. In Ethiopia, we worked exclusively with female CHWs who are paid government workers with an average of 10 years of formal schooling and resident in their catchment area. In Malawi, we worked with approximately equal numbers of male and female CHWs who were each paid health workers with approximately 10 years of schooling, but sometimes not a resident in their catchment area. In Mali, we worked with community volunteers who were resident in their local community and had been nominated by it. More than 70% of these volunteers were female and had no formal education.

**Table 2:** Design and Implementation Characteristics of RMM community-based methods tested in Ethiopia, Malawi, and Mali.

Country	CHARACTERISTICS OF THE COMMUNITY WORKER							CHARACTERISTICS OF THE RMM METHOD BEING TESTED		
	Type of worker; primary duties other than RMM	% Female	No. of Workers	Mean monthly worker remun- eration (in 2013 US$)	Mean years of schooling	Resident Population per CBHW	% Resident in Catchment Area	Incentives		Mean frequency of RMM supervision per month
								Financial	Other	
**Ethiopia**	Health Extension Worker (HEW); preventive/ curative health care & promotion	100%	183	~$35	≥10th Grade	2,799	100%	Monthly transportation allowance of ~$12	Backpacks; initial training	Monthly
**Malawi (Phases I & II)**	Health Surveillance Assistant (HSA); preventive/ curative health care & promotion	~50%	160	~$100	10 years plus 10 weeks HSA training	1,273	<50%	Quarterly airtime for phone calls, periodic data review meeting,data review meeting participation allowance ($11/meeting)	Both Phases: Village Health Register, cellphone, backpack, ≥ 1 data review meeting. Phase II only: text messages, RMM “HSA of the Quarter” incentive, regular data review meetings every 3 months.	Monthly
**Mali**	Lay volunteer health works (Relais); health promotion	28%	78	Volunteer	26% have any formal education*	412	100%	$10/month incentive plus $2 in airtime/month	Registers, quarterly meetings (provide lunch, pay for transportation)	Monthly

In all three study areas, supervision of the vital events reporting work performed by CHWs was undertaken on a monthly basis and CHWs were provided with monthly transportation allowances and cell phone airtime. In Malawi, we undertook a midterm evaluation of the RMM community-based vital events reporting by CHWs. Based on the results of this assessment, we instituted more regular data review meetings, introduced Short Message Service (SMS) reminder and guidance messages, and launched a quarterly award for the best performing CHW. These post-assessment incentives and their associated effects in Malawi are discussed in detail in Joos et al. (2015) [24].

### Implementation of community-based vital events reporting

The RMM team in each country prepared for implementation by developing standard procedures for catchment-area mapping, recording and reporting of vital events and consistent approaches to supervising their application. The team trained community-based workers and their supervisors, and developed standard operating procedures for data reporting and cleaning. We used a trial period of three months to finalize these procedures in each country site.

Each method was implemented for a period of at least fifteen months, with careful documentation of processes and costs. Intermediate assessments of the procedures and data quality were conducted at least once in each setting, and used as a basis to **reinforce performance and operations.**

#### Validation data

We used current best practices for child mortality measurement in countries without functioning reliable vital registration system, consisting of household surveys with full birth or full pregnancy history from women of reproductive age. During full birth history interviews, women of reproductive health are asked to list all their children ever born with date of birth and survival status, including age at death for children who have died. For pregnancy history interviews, women are asked about all pregnancies they ever had. These approaches are used to estimate child mortality retrospectively for periods of up to 25 years preceding the survey [25]. In our RMM study areas in Mali and Malawi, we carried out endline data collection using a full pregnancy history instrument in all households in Mali and in a random sample of 10,000 households in Malawi [23, 24]. Each of the 160 CHW catchment areas in Malawi was survey with a sample size of households proportional to the total number of households in the catchment areas. In Ethiopia, the endline validation survey included a full birth history of 28,000 households, building on an existing data collection design for an evaluation study of a child health strategy in the same RMM areas [22, 26]. The survey in Ethiopia used two stage cluster sampling in the two zones of the RMM study area. The first stage of sampling involved census enumeration areas that were sampled probability proportional to size. Households to be interviewed were then sampled at a second stage using a full listing of all households in each sampled enumeration area. The quality of all the validation data was assessed using standard demographic data quality assessment methods [22, 23, 24].

### Analysis

We used an array of standard metrics to assess each of the community-based RMM methods across the country settings. *Process metrics* focus on the completeness of reporting of vital events, obtained by comparing the numbers of births and deaths reported through the RMM method to those estimated from the best practice survey or census. *Data quality metrics* are those commonly used in demographic analysis, and include the sex ratio at birth, the ratio of neonatal to infant deaths, the ratio of infant to under-five deaths, and the distributions of under-five deaths by age in months and neonatal deaths by age in days. *Accuracy metrics* include the concordance between the neonatal, infant and under-five mortality rates produced by the RMM method and the current best practice method. In comparisons of accuracy across country settings, we use average annual ratios of under-5, infant, and neonatal mortality rates. We annualized these ratios to improve comparability across the three country sites. Throughout the analysis we explicitly consider both the performance of the RMM method and possible shortcomings in the current best practice method, defined as full pregnancy or full birth histories collected via household surveys or censuses.

**Fig 1:**
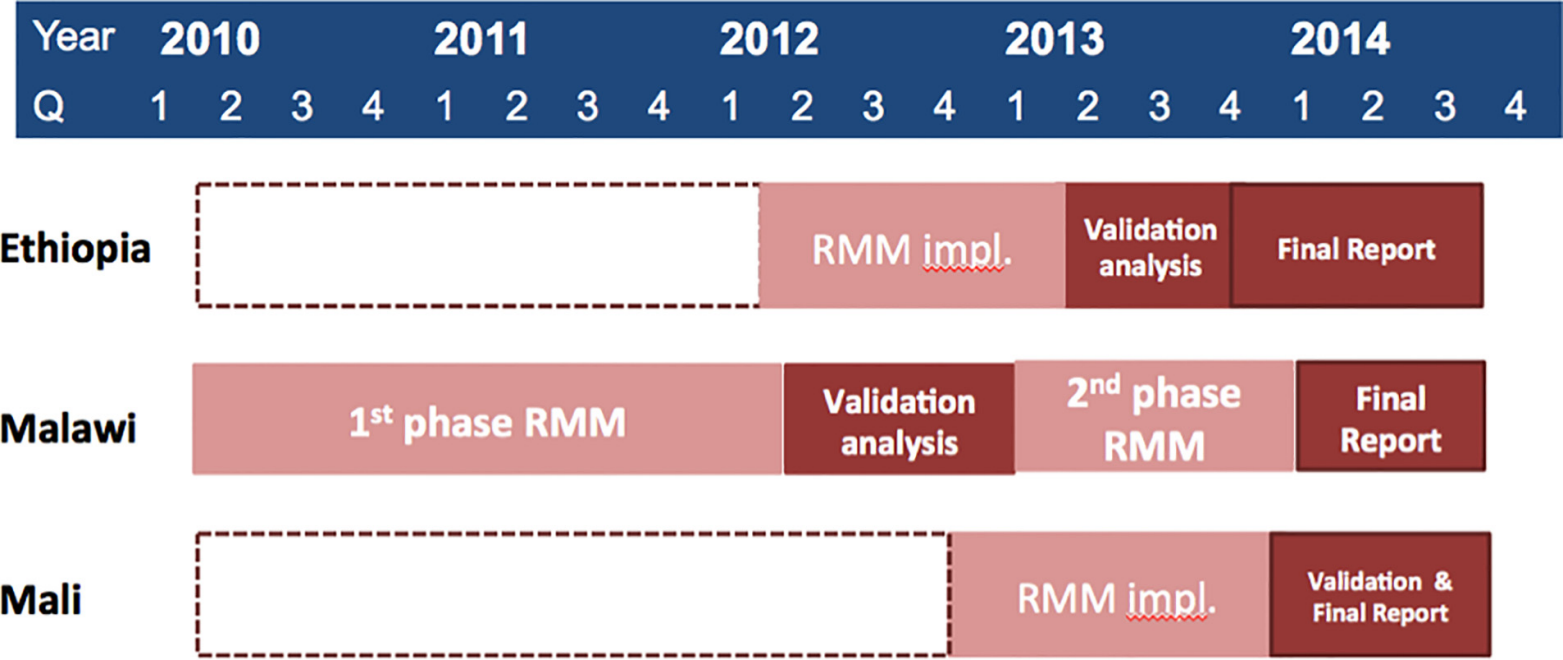
Implementation Timeline of RMM Community-based Method by Country Setting.

Data on births and under-five deaths reported by CHWs for the periods of January 2012 to March 2013 in Ethiopia, January 2010 to December 2013 in Malawi, and July 2012 to September 2013 in Mali were included in the validation analysis, as shown in Fig 1. We analyzed the data for rolling 12-month periods, with starting periods differing by exactly three months. Data analysis was conducted in R [27].

We calculated the number of births and neonatal, infant, and under-five deaths reported by CHWs for each 12-month period. We did not adjust the CHW vital events data for missing monthly reports, given that there were only a small number of missing reports in the Ethiopia and Malawi study sites, and none in Mali. We calculated neonatal, infant and under-five mortality rates for each period by dividing the number of these deaths documented by CHWs in a given period by the total births documented by CHWs in that same period.

The validation analysis involved two components: (1) an evaluation of the completeness of births and deaths reporting by CHWs; and (2) a comparison of under-five, infant, and neonatal mortality rates calculated from the CHW data with those estimated from the FPH validation data for each 12-month period. We calculated mortality rates from the CHW data and the validation survey data in the same way to ensure direct comparability.

To evaluate the completeness of births and under-five deaths documented by the CHWs, we estimated the expected number of births and under-five deaths that should have been collected by CHWs for each 12-month period. We estimated the crude birth rate and the under-five mortality rate in each period directly from the validation data. To estimate the expected number of births that should have been reported by CHWs, we multiplied the crude birth rate for each 12-month period by the total population size of the RMM catchment area in each district. The expected number of under-five deaths was calculated similarly, by multiplying the under-five mortality rate estimated from the validation data by the expected number of births (calculated as described above). We examined the completeness of births and under-five deaths data collected by CHWs by calculating the ratio of the total numbers of births and under-five deaths documented by CHWs to the expected numbers estimated as described above.

Each country team prospectively tracked costs associated with the community-based RMM methods. We reviewed all costing data centrally, and revised costs in collaboration with the RMM country teams. We inflated costs using local consumer price indices and converted to 2014 United States Dollars. For each method we calculated total cost, average annual cost per 1,000 population, and cost per vital event reported. We excluded the costs of the validation surveys from analysis as these would overestimate the true cost of RMM.

## Results

Table 3 presents the standard metrics for process and accuracy of the RMM community-based method as implemented in the three study countries, along with the average annual running cost per 1,000 population.

**Table 3:** Summary of process, accuracy and cost results for RMM methods tested.

Country	Type of Worker	PROCESS	*Births*	ACCURACY RELATIVE TO CURRENT BEST PRACTICE (Average Annual Ratio RMM:Best Practice Census or Survey)				AVERAGE ANNUAL RUNNING COST PER 1,000 POPULATION(US$)
		*Average % of catchment areas reporting per month*		*Under-five Deaths*	*Neonatal mortality rate*	*Infant mortality rate*	*Under-five mortality rate*	
Ethiopia (15 months, Jan 2012 –Mar 2013)	Health Extension Worker (HEW)	95.7	30.1	21.7	89.0	81.0	72.0	$ 523
Malawi (48 months, Jan 2010 –Dec 2013)	Health Surveillance Assistant	96.6	65.9	50.6	74.0	67.5	67.7	$434
Mali (15 months, Jul 2012 –Sep 2013)	Community health volunteer (Relais)	100	90.3	90.8	128.8	120.0	100.6	$6,344

The RMM community-based workers submitted their reports regularly, suggesting that the process of timely vital events reporting by CHWs across diverse settings is feasible. The average percent of catchment areas for which monthly reports were submitted was over 95% in Ethiopia and Malawi and 100 percent in Mali.

**Fig 2:**
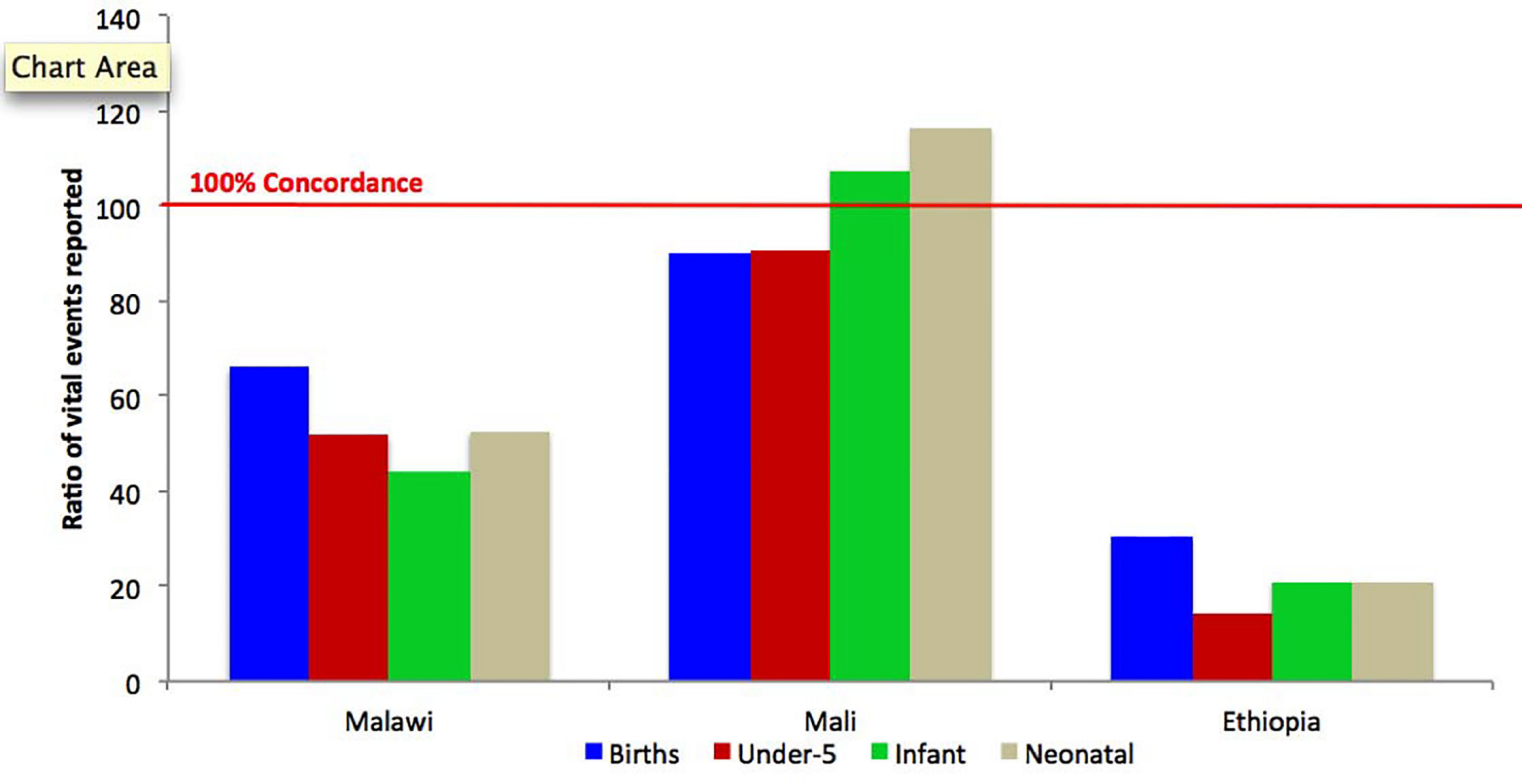
Reporting Completeness of Vital Events Information by CHBWs relative to the expected number of events estimated from full pregnancy histories collected through a sample survey or census

The completeness of the community-based reports, relative to the best-practice FPHs, varied widely by country, and to a lesser extent, by type of event within countries. In Ethiopia and Malawi, as shown in Fig 2, the concordance between community and best practice reports was higher for births than for under-five deaths. CHWs severely under-reported under-five deaths in both Ethiopia and Malawi, but not in Mali. There was higher concordance for neonatal deaths than under-five deaths in Ethiopia and Malawi, and higher proportions of neonatal deaths documented by CHWs than reported in the best practice FPHs in Mali.

**Fig 3:**
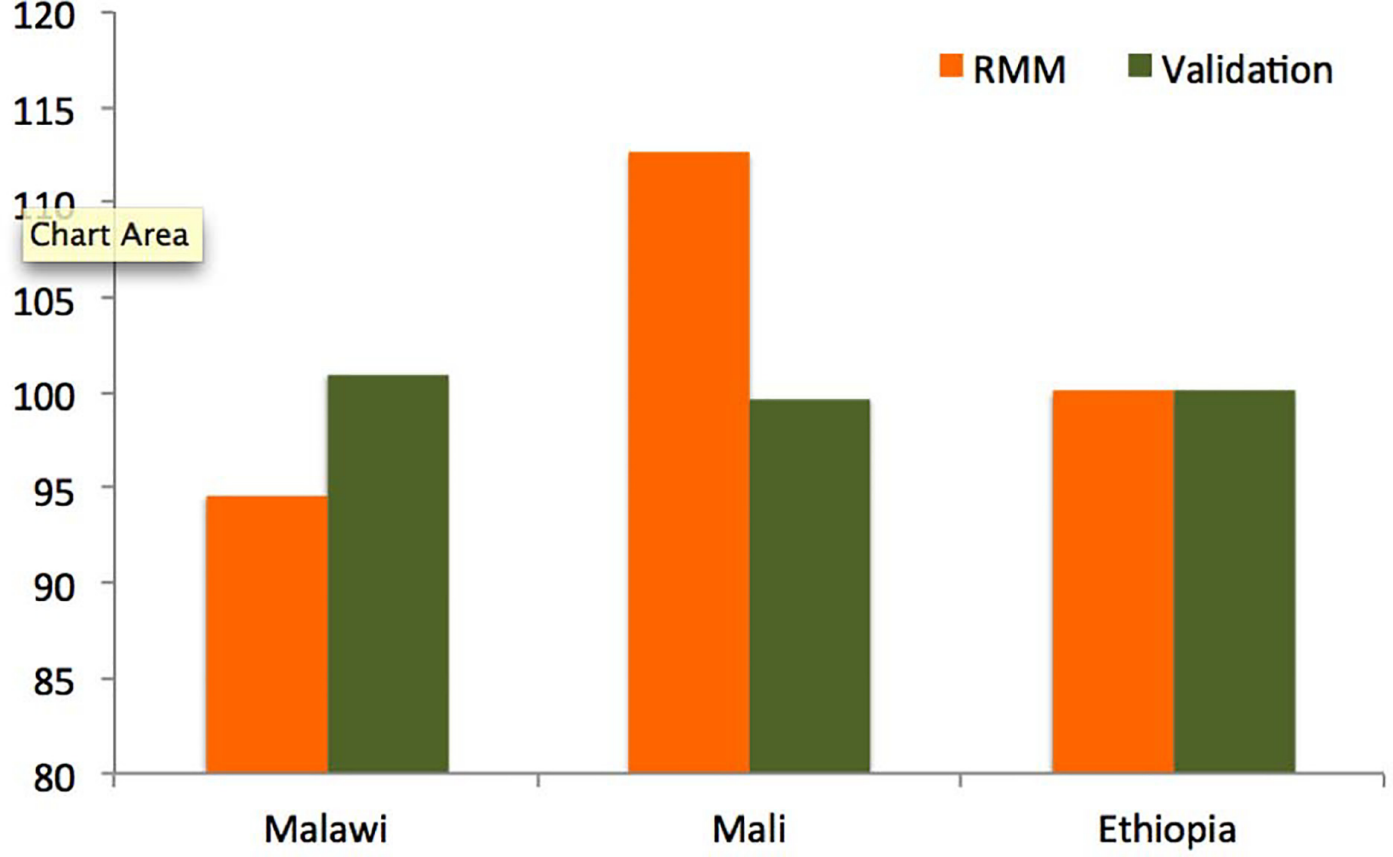
Average sex ratio at birth for RMM data collected by community-based health workers compared with those from comparable full pregnancy histories for annualized periods

The results on data quality are presented for annualized periods in Fig 3 and Table 4. Fig 3 shows the average sex ratio at birth for the RMM data collected by CHWs, compared with those from comparable full pregnancy histories. The sex ratios at birth for annualized 12-month periods are, on average, consistent between the two data sources. In Mali, the CHWs documented a higher proportion of male births to female births than was observed in the corresponding FPHs, but was still well within 20% of that documented by the FPHs. Table 4 shows mortality ratios (the ratio of neonatal deaths to infant deaths and the ratio of infant deaths to under-five deaths) for the two data sources. Vital events reporting by CHWs documented higher proportions of infant deaths within the neonatal period than the FPHs across all three country settings, with an average difference of 6 percentage points (range 3.9 in Mali to 9.6 in Malawi). Differences between the two methods were larger for the proportion of infant deaths to under five deaths, with an average absolute difference of 9 percentage points (range 7.8 in Malawi to 9.4 in Mali), and the difference in ratios in Ethiopia and Mali was positive while in Malawi was negative. In the FPH data that we used to validate CHW-based reporting of vital events, we observed digit preference (commonly referred to as “age heaping” in demography) for ages ending in 0 and 5.

**Table 4:** Average number of neonatal, infant, and under-five deaths documented by community workers and average annualized death ratios based on community-based health worker reports and full pregnancy histories (FPHs) collected via surveys and censuses.

Country	Average number of annualized deaths documented by community-based health workers			Ratio of average annualized deaths based on RMM reports to associated death ratios based on best practice FPHs			
				Neonatal:Infant		Infant:Under-five	
	Neonatal	Infant	Under-five	RMM	FPH	RMM	FPH
**Ethiopia**	125	188	244	66.5	61.0	77.0	68.4
**Malawi**	87	142	265	61.2	52.6	53.5	61.3
**Mali**	53	106	179	50.0	46.1	59.2	49.8

In Ethiopia and Malawi we conducted home visits to confirm that the CHWs recorded the events they identified correctly and completely in their registers. In Malawi, the proportions of births and deaths reported by CHWs among a sample of 2,227 (14%) birth events and 646 (59%) death events that were independently verified in village health registers (VHR) were 87% and 89%, respectively; in Ethiopia the proportion of births and deaths reported by CHWs among a sample of 476 (8%) birth events and 139 (47%) death events that were independently verified in family folders maintained at local health posts was 93% and 91%, respectively [22, 25]. In both Ethiopia and Malawi, the consistency of recording of event dates was around 90% for the vital events documented by both CHWs and in VHRs or family folders. In Mali we attempted to match individual events between the RMM and FPH methods to allow a more in-depth understanding of error patterns, with limited success. [23].

**Fig 4:**
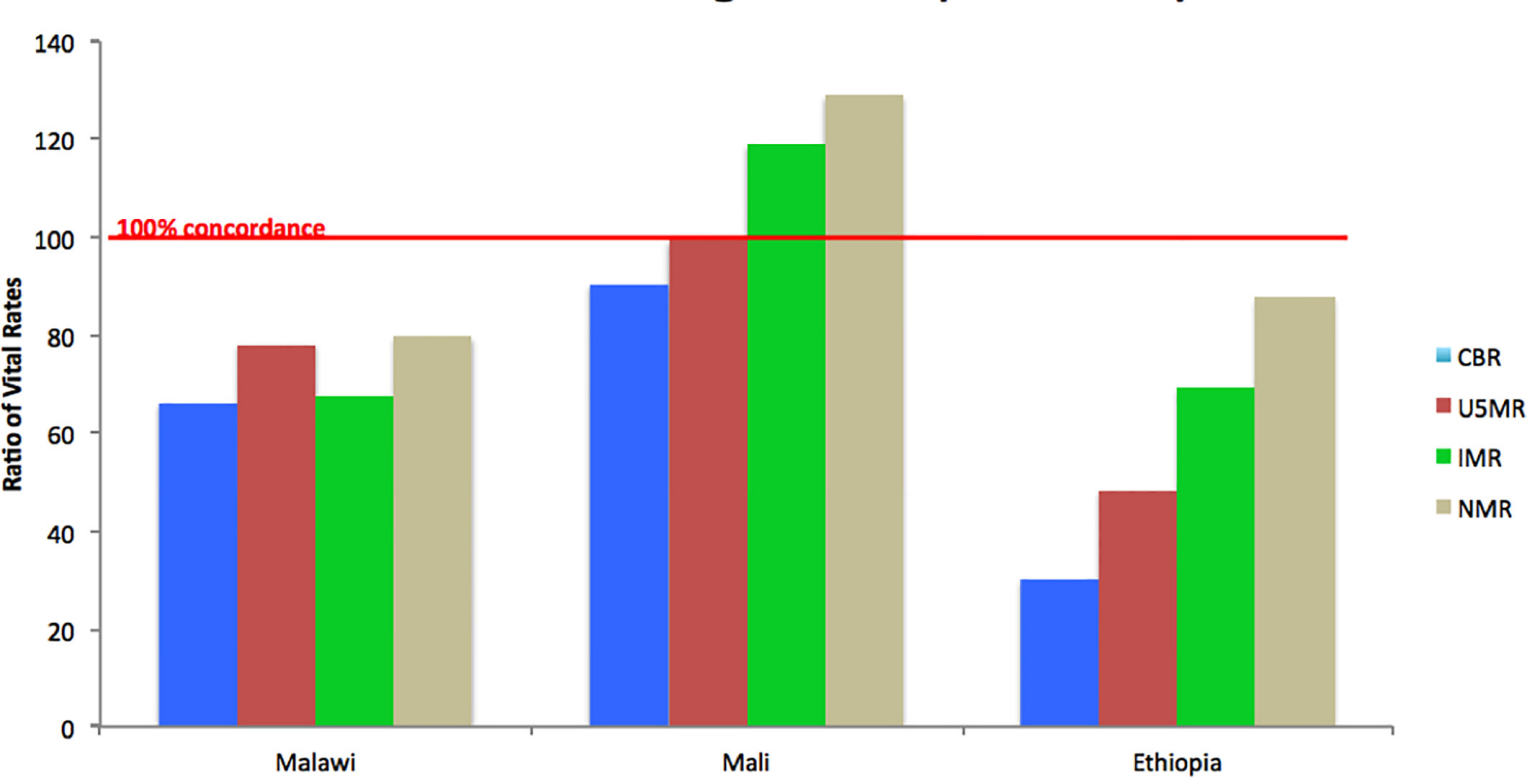
Accuracy of Vital Rate Estimates using RMM community-based method in each setting, compared to estimates derived from full birth histories collected through a sample survey or census

Fig 4 presents results on the accuracy of vital rate estimates produced by the community-based method in each setting, compared to the estimates derived from the FBHs. We present concordance of the RMM methods tested in these three countries, as reflected in the ratios of crude birth rates and mortality rates vital events reported by CBWs relative to the corresponding rates estimated through best practice surveys or censuses, Mali performed the best, followed by Malawi and Ethiopia. In Mali, the accuracy of the RMM method was above 90% for the crude birth rate (CBR) and U5MR, but well over 100% for the IMR and the NMR. The pattern is similar in Ethiopia, but with extreme underreporting of the CBR and U5MR.

The RMM programs in Ethiopia, Malawi, and Mali cost $444,392, $353,782, and $288,766, and had average annual costs per 1,000 population of $523, $434, and $6,344, respectively. These amounted to $72, $21, and $149 per vital event reported in Ethiopia, Malawi, and Mali, respectively. Major cost categories included central office salaries, supervision of CHWs, and costs of training and equipping CHWs for RMM.

## Discussion

The RMM project has generated important information about the feasibility and accuracy of CHW-based approaches to measuring changes in under-five mortality within periods of 12–24 months. The approaches tested varied widely, as did the study settings, and the results must therefore be generalized with care. The scope of the study also did not permit a full assessment of the effects of the health system context on the performance of each method. The accuracy of the results produced by the RMM community-based method was tested against a well-known and established population-based method for mortality estimation–namely, full pregnancy histories or full birth histories collected via household surveys or censuses.

Mali demonstrates that complete and accurate reporting of vital events by community workers is possible, but the investments of resources to support locally-defined incentive structures and levels of supervision and monitoring that were used in the RMM study [23] are unlikely to be feasible for implementation at scale and over time.

In Ethiopia and Malawi, the RMM community-based method under-estimated the annualized under-five mortality rate by more than 20%. Whereas, in Mali the two methods produced annualized under-five mortality rate estimates that were statistically equivalent. Our Mali findings demonstrate that, with relatively strong supervision and strong community engagement with CHWs, community-based workers were capable of collecting routine vital events reports that facilitate the accurate tracking of changes in under-5 mortality rates on an annual basis. Our findings from Ethiopia and Malawi are notably more modest–indicating that task shifting of CHWs and assignment of CHWs to large geographic areas (as was the case in Ethiopia) as well as frequent turnover of staff (as was the case in Malawi) can be serious impediments in the estimation of accurate annualized under-five mortality rates using community-based vital events reporting by CHWs.

Validating the RMM methods in low-income settings is challenging. The vital registration systems used as a “gold standard” in some high-income countries have rates of completeness that meet the 90% standard recommended by the United Nation Statistics Division, and even the results from these systems are adjusted and refined to account for content and coverage errors using censuses and surveys [28]. In most low- and middle-income countries, vital registration systems are weak and incomplete, and population censuses and household surveys are usually the only available source of data that can be used to estimate vital rates. Although we have used censuses and surveys as current best practice in the RMM work to validate the estimates produced by community-based vital event reporting methods, we recognize that these methods do not provide a “gold standard” and are subject to a number of limitations and challenges associated with retrospective reporting by the mother rather than direct observation and enumeration at the time of the event.

We found that both the RMM community-based method and the FPHs produced vital events data that are generally consistent with known patterns, but with some exceptions. The observed sex ratios at birth, which are expected to fall between 102 and 107 male births per 100 female births in most populations [29], indicated some underreporting of female births relative to male births, particularly in Ethiopia and Malawi (Fig 3). We do not know whether these thresholds are true for the RMM study population. In Mali, the CHWs documented, on average, higher sex ratios at birth than was documented in the corresponding FPH. We also saw indications of age heaping for age at death in the FPHs in particular, especially at 12 months of age. This phenomenon is common in retrospective mortality surveys in low resource settings, where the mother may not be able to recall the child’s exact age at death with precision. Notwithstanding these issues, the completeness and the quality of the vital events data collected in Mali were sufficiently high to allow for a valid test of the performance of the community-based method in estimating mortality over 12 month periods.

In each of the RMM settings that implemented a community-based vital events reporting system, we analyzed the ratios of early neonatal to neonatal deaths and neonatal deaths to infant deaths as one measure of data quality. In all three RMM settings, the ratios of early neonatal to neonatal deaths and neonatal deaths to infant deaths were larger than expected based on current best estimates derived from household survey interviews, as shown in Table 4. This suggests that community-based methods may be more effective than the full birth histories conducted during household surveys or censuses in capturing neonatal deaths. There is no one simple reason for this. One factor may be that in the RMM community-based methods tested here we tracked pregnancies as well as births and deaths, and in Mali, proactively reminded the CHWs to follow up pregnant women around the expected time of birth. Another factor, at least in Malawi and Ethiopia, may be that the CHWs also had responsibility for providing pregnant women with advice on preparations for deliveries. Yet another may be that in settings where the CHWs were selected with inputs from community members, as in Mali, they were more closely connected to their communities and therefore more likely to be aware of, or trusted with information about, early neonatal deaths. However, it is also possible that our RMM community-based methods were susceptible to misclassifying some stillbirths as neonatal deaths. Further investigation, using full pregnancy histories and the routine reporting of pregnancies by community-based health workers, is needed to evaluate whether the higher proportions of neonatal deaths suggest improved reporting of early deaths or rather potential misclassification of stillbirths (as neonatal deaths) by CHWs.

The challenges associated with implementing community-based vital reporting systems varied by setting. In Ethiopia, the CHWs charged with vital event reporting were often called away from their catchment areas for other duties. In Malawi, there were high rates of turnover among CHWs, requiring frequent refresher training. We found that the introduction of customized worker incentives intended to improve the completeness and quality of vital events reporting in Malawi were insufficient to counter the effects of staff turnover [24]. Perhaps, most notably there was considerable variation in the size of the resident population per CHW as shown in Table 2. In Ethiopia, each CHW was responsible on average for 2,799 residents in their local area, compared with 1,273 residents in Malawi, and the 412 in Mali. This amounts to a notable variation in workload for individual CHWs that directly affects the completeness of vital events reporting and therefore the accuracy of resulting child mortality rate estimates based on CHW routine reports. No one-size-fits-all approach will be successful in guaranteeing complete and accurate reporting of vital events by community-based health workers.

Costing estimates varied greatly by country. Malawi had the lowest cost per vital event reported and the lowest average annual cost per 1,000 population. Ethiopia had the most expensive program but a similar cost per 1,000 population to Malawi. Mali had the highest average annual cost per 1,000 population and the highest cost per vital event reported, which was double that of Ethiopia and seven times that of Malawi. An important question is the extent to which the success of the RMM approach in Mali is attributable to greater investment and smaller scale relative to the other sites. Further attempts to cost community-based tracking of vital events should give careful consideration to country and program-specific factors from an early stage.

The CHW reporting methods evaluated in this study were defined in collaboration with Ministries of Health and local partners in each setting, based on the existing health system structure and human resources. There are other approaches for generating vital statistics that may be more promising in some settings, and warrant careful assessment. For example, Malqvist and colleagues used a combination of key informant interviews, questionnaires and health facility records in Vietnam to ascertain neonatal deaths, but similar to our findings they reported high levels of under-reporting [30]. Advances in information technology may provide new options for community-based reporting [31, 32], but will require rigorous evaluation. Furthermore, AbouZahr et al. (2015) have noted that innovations in information technology are not sufficient to guarantee improvements in CRVS systems, rather a strong integrated program logic that incorporates innovations in new technology needs to drive system improvements [33].

## Conclusions

Our findings from Ethiopia, Malawi and Mali indicate that the completeness and accuracy of vital events reporting by CHWs is affected by a number of operational factors. Perhaps most surprisingly, vital events reporting by unpaid community-based volunteers in Mali was more accurate than that by paid workers in Malawi and Ethiopia. This finding underlines the importance of community acceptance and engagement with the vital events reporting systems and acceptance of community health workers, as well as the importance of manageable workloads, effective supervision and continuous quality control procedures. As the international community seeks to substantially scale-up civil registration and vital statistics systems as part of the post-2015 development agenda, the need for customized approaches to improving vital events reporting systems that build on existing systems should not be overlooked. In contemporary rural sub-Saharan Africa, relatively few deaths occur in health facilities and thus health facility records provide an inadequate basis to monitor population-level mortality dynamics [6]. For most of the rural population in low-income countries, community-based health workers are the first and most common interface with the health system–particularly when it comes to reproductive health and maternal, newborn and child health. Our results from Mali indicate that, at least at small scale, RMM community-based methods can generate complete vital events reporting and accurate annualized child mortality indicators. However, our results from Ethiopia and Malawi highlight important challenges in field implementation, supervision and reporting completeness. This suggests that those advocating for drawing on CHWs to advance vital events reporting and improved child mortality measurement should be cautious and that further research is needed to understand how to enhance existing CHW capacity, streamline field processes across diverse low-resource settings, and further unpack the trade-offs between non-sampling errors (such as recall errors) associated with FPHs and FBHs and CHW-based ‘real-time’ reporting problems (such as disclosure bias) of vital events.
